# Degradation of Glucan Primers in the Absence of Starch Synthase 4 Disrupts Starch Granule Initiation in Arabidopsis*

**DOI:** 10.1074/jbc.M116.730648

**Published:** 2016-07-25

**Authors:** David Seung, Kuan-Jen Lu, Michaela Stettler, Sebastian Streb, Samuel C. Zeeman

**Affiliations:** From the Institute for Agricultural Sciences, ETH Zurich, 8092 Zürich, Switzerland

**Keywords:** Arabidopsis thaliana, carbohydrate metabolism, chloroplast, photosynthesis, plant biochemistry, alpha-amylase, starch biosynthesis, starch granule initiation, starch synthase

## Abstract

*Arabidopsis* leaf chloroplasts typically contain five to seven semicrystalline starch granules. It is not understood how the synthesis of each granule is initiated or how starch granule number is determined within each chloroplast. An *Arabidopsis* mutant lacking the glucosyl-transferase, STARCH SYNTHASE 4 (SS4) is impaired in its ability to initiate starch granules; its chloroplasts rarely contain more than one large granule, and the plants have a pale appearance and reduced growth. Here we report that the chloroplastic α-amylase AMY3, a starch-degrading enzyme, interferes with granule initiation in the *ss4* mutant background. The *amy3* single mutant is similar in phenotype to the wild type under normal growth conditions, with comparable numbers of starch granules per chloroplast. Interestingly, the *ss4* mutant displays a pleiotropic reduction in the activity of AMY3. Remarkably, complete abolition of AMY3 (in the *amy3 ss4* double mutant) increases the number of starch granules produced in each chloroplast, suppresses the pale phenotype of *ss4*, and nearly restores normal growth. The *amy3* mutation also restores starch synthesis in the *ss3 ss4* double mutant, which lacks STARCH SYNTHASE 3 (SS3) in addition to SS4. The *ss3 ss4* line is unable to initiate any starch granules and is thus starchless. We suggest that SS4 plays a key role in granule initiation, allowing it to proceed in a way that avoids premature degradation of primers by starch hydrolases, such as AMY3.

## Introduction

Plants store carbohydrates in the chloroplast as semicrystalline starch granules. In *Arabidopsis* mesophyll cells, 5–7 starch granules are typically produced in each chloroplast during the day (1). The degradation of these granules into soluble sugars at night provides energy for growth in the absence of light.

Starch contains two distinct polymers, amylopectin and amylose, both solely containing glucose subunits. Amylopectin has a branched tree-like structure, with glucosyl units linked via α-1,4-bonds into linear chains. These linear chains are joined together via α-1,6-bonds at branch points, which occur on average every 20–25 glucose units (2). Amylose is virtually unbranched, being composed of long linear chains of α-1,4-linked glucose units. Crystallinity in the starch granule arises from the secondary structures of amylopectin, where pairs of adjacent chains form double helices that pack tightly together. The formation of crystalline granules distinguishes starch from glycogen, a closely related storage polymer in bacterial, fungal, and animal cells. Although glycogen also contains α-1,4 linear glucan chains with α-1,6 branches, the branch points occur more frequently (on average every 12 residues). The resulting structure does not allow crystallinity, and thus glycogen remains soluble (3).

The synthesis of both amylopectin and amylose is mediated by starch synthases (SSs),^2^ a specialized set of glucosyltransferases closely related to glycogen synthases (4). These SSs elongate glucan chains by transferring the glucose moiety from ADP-glucose onto acceptor glucans in the α-1,4 configuration. In *Arabidopsis*, there are five classes of SS (SS1, SS2, SS3, SS4, and GBSS). GBSS is specialized for the synthesis of amylose, and *Arabidopsis* mutants lacking GBSS produce amylose-free starch (5). SS1, SS2, and SS3 are involved in amylopectin synthesis, and mutants lacking any of these isoforms have altered amylopectin structure (6–8). Branching enzymes and debranching enzymes are necessary for establishing the typical branch structure of amylopectin (9, 10).

Despite our extensive knowledge of the biochemistry underlying starch synthesis, some important processes remain poorly understood. In particular, we do not yet understand how starch granule synthesis is initiated. Glycogen synthesis in mammals is initiated by a specialized protein, glycogenin, which self-glucosylates and serves as a primer for glucan synthesis (3, 11). Plants have proteins with similarity to glycogenins, but their role in starch synthesis remains unclear (12). There is, however, strong evidence that SS4 plays a critical role in starch granule initiation in *Arabidopsis* leaves. SS4 contributes very little to the measurable starch synthase activity and does not have a major influence on amylopectin structure (13). However, mutants lacking SS4 have drastically reduced granule number per chloroplast, initiating one or at most two granules, rather than the usual 5–7. Mutations in each of the other SSs are not reported to result in altered granule number, and even *ss1 ss2 ss3* triple mutants have normal numbers of granules per chloroplast, despite having drastically reduced starch content (14). This suggests that SS4 has a specialized role among SSs in granule initiation. Because *ss3 ss4* mutants are completely starchless (14), it appears SS3 can initiate some granules in the absence of SS4, but with poor efficiency. It is hypothesized that only SS4 and to a lesser extent SS3 can generate glucan primers that the other SSs require as a substrate to synthesize a starch granule (15). Without this primer, the other starch synthases remain almost inactive, resulting in the accumulation of ADP-glucose (15, 16). This in turn has a detrimental effect on plant growth; the sequestration of adenylates into ADP-glucose in chloroplasts is proposed to limit the ADP available for photophosphorylation, explaining why *ss4* and *ss3 ss4* mutants have reduced growth and pale phenotypes (16).

Here we show that the granule initiation process must be regulated in a way that prevents interference from starch degradation enzymes. Although starch is primarily degraded at night in *Arabidopsis* leaf chloroplasts, enzymes involved in starch degradation are also present during the day (17, 18). The redox regulation of some starch degradation enzymes also suggests that they could potentially be activated by light (19). We initially observed that the activity of an α-amylase, AMY3, is greatly reduced in *ss4* and *ss3 ss4* mutants. Remarkably, the complete elimination of AMY3 activity in *ss4* and *ss3 ss4* backgrounds increased the number of starch granules per chloroplast and a greatly improved growth phenotype in both mutants. Our results demonstrate that AMY3 can interfere with the granule initiation process and provide greater insight into the role of SS3 and SS4 during granule formation.

## Results

### 

#### 

##### AMY3 Protein Abundance Is Dramatically Reduced in the ss4 Mutant

**FIGURE 1.**
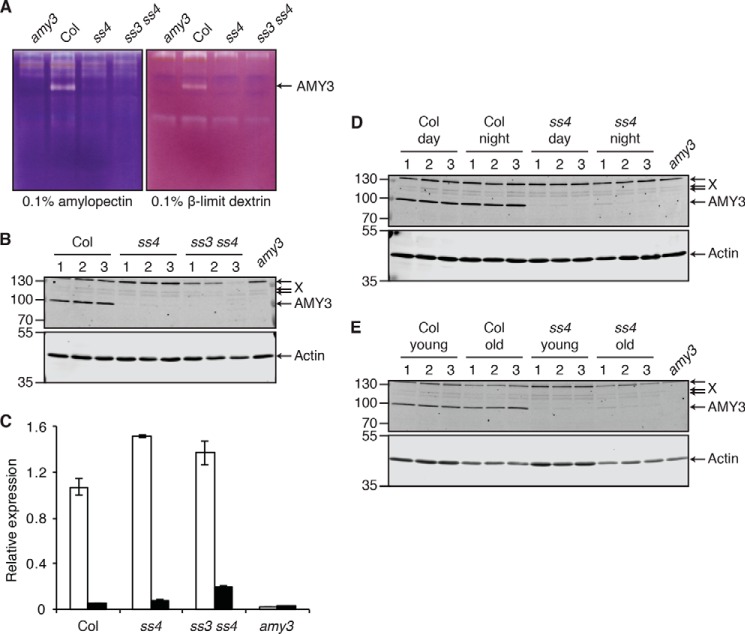
**AMY3 abundance is reduced in *ss4* and *ss3 ss4* mutants.** *A*, activity of starch-degrading enzymes on native PAGE gels containing 0.1% (w/v) amylopectin or β-limit dextrin. Soluble proteins were extracted from *Arabidopsis* leaves harvested 4 h into the day and loaded onto gels on an equal fresh weight (1.8 mg) basis. The gels were incubated in activity medium after electrophoresis. The bands were observed by staining with iodine/potassium iodide solution. *B*, immunoblot detection of AMY3 in soluble protein extracts from leaves harvested 4 h into the day. The *numbers* represent the three biological replicates harvested for each genotype. The gels were loaded on an equal fresh weight (0.9 mg) basis. AMY3 and actin (as a loading control) were detected concurrently on the same membrane using secondary antibodies conjugated to different infrared fluorescence dyes (800CW for AMY3 and 680RD for actin). AMY3 and actin bands, as well as three unidentified bands that cross-react with the AMY3 antibody (*X*), are indicated with *arrows. C*, AMY3 transcript levels at the end of day (*white bars*) and end of night (*black bars*) as measured by quantitative RT-PCR. Expression was calculated relative to the reference gene, *YLS8*. The values represent the means ± S.E. from *n* = 3–4 individual rosettes. *D*, same as *B*, except comparing extracts from leaves harvested during the day (4 h into day) with those harvested during the night (2 h before dawn). Note that samples for day are the same as those analyzed in *B. E*, same as *B*, except comparing extracts from young leaves with those from old leaves. The leaves were harvested 4 h into the day. Note that samples for young leaves are the same as those analyzed in *B*.

To assess the involvement of starch degradation enzymes in granule initiation, we first investigated whether the activities of starch-degrading enzymes were altered in *ss4* and *ss3 ss4* mutants. Total soluble proteins extracted from young rosette leaves were separated on native PAGE gels containing amylopectin or β-limit dextrin as substrates. Enzymatic activities that degrade or modify these substrates were visualized by staining the gels with iodine. The intensities of most activity bands observed in the *ss4* or *ss3 ss4* mutants were not different from those observed in the wild type (Fig. 1*A*). However, we observed a striking reduction of an activity that could degrade both substrates. A previous study identified this band as the activity of the chloroplastic α-amylase AMY3 (20). This band was absent in protein extracts from the *amy3* knock-out mutant, reconfirming this identification (Fig. 1*A*).

We then investigated whether the diminished AMY3 activity in *ss4* and *ss3 ss4* resulted from changes in AMY3 protein and/or transcript abundance. Proteins were extracted from young rosette leaves harvested 4 h into the day, and AMY3 abundance was examined by immunoblotting with AMY3-specific antibodies (17). Protein extracts from *ss4* and *ss3 ss4* mutants contained far less AMY3 protein than extracts from the wild type (Fig. 1*B*). Quantification of the fluorescence signal showed that AMY3 band intensity in *ss4* was on average 2.5 ± 0.9% of that observed in the wild type. AMY3 bands were very faint in *ss3 ss4* and could not be accurately quantified. We used quantitative RT-PCR to determine whether these changes in AMY3 protein abundance resulted from alterations in *AMY3* transcription. *AMY3* transcript levels fluctuate diurnally, with peak expression at the end of day and low expression at the end of night (17, 18). We observed this diurnal change in *AMY3* transcript level in both the *ss4* and *ss3 ss4* mutants (Fig. 1*C*). Interestingly, there was slightly more transcript in the mutants than the wild type at both the end of day and the end of night. Thus, *ss4* and *ss3 ss4* mutants have less AMY3 protein than the wild type because of changes at the post-transcriptional level.

**FIGURE 2.**
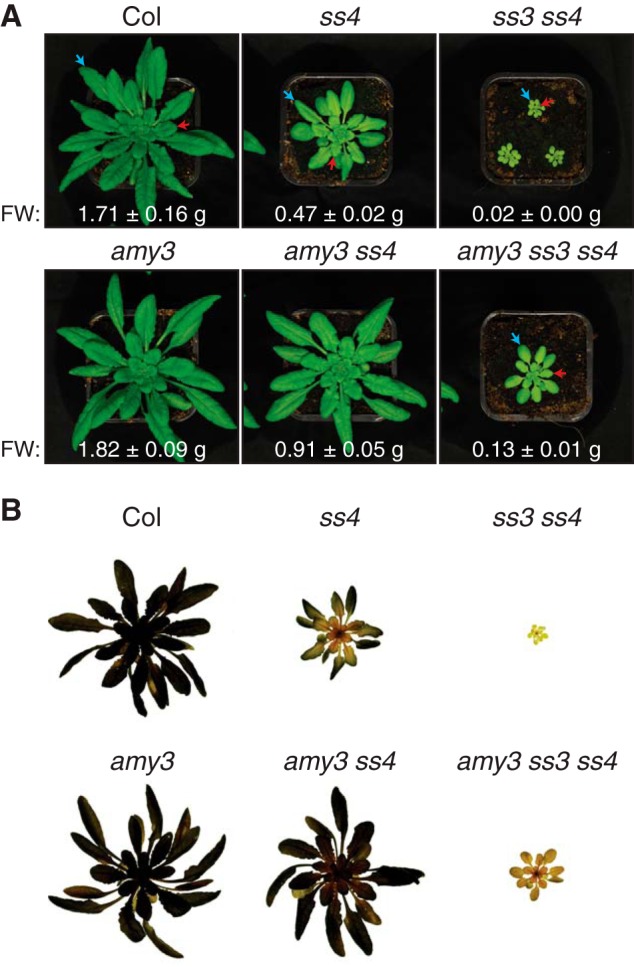
**Loss of AMY3 suppresses the *ss4* and *ss3 ss4* phenotypes.** *A*, representative rosettes of 4-week-old plants were photographed. The *numbers* represent the mean fresh weight (*FW*) ± S.E. (*n* = 8). *Red arrows* indicate leaves representative of those defined as “young” leaves, whereas *blue arrows* represent “old” leaves. *B*, visualization of starch in tissues by staining with iodine solution. Representative rosettes of 4-week-old plants were cleared of chlorophyll in 80% (v/v) ethanol prior to staining. *Dark staining* indicates the presence of starch.

Similar changes in AMY3 abundance could be observed in *ss4* leaves harvested during the night (Fig. 1*D*). Additionally, we tested whether AMY3 abundance was influenced by leaf age in the wild type and *ss4*, because the *ss4* mutant contains more starch in older leaves than in young leaves (15). Examples of young and old leaves are indicated in Fig. 2*A*. AMY3 band intensity in extracts from old leaves were not significantly different from those in young leaves (Fig. 1*E*). Taken together with the native PAGE gels, these data suggest that the loss of SS4 results in a pleiotropic reduction of AMY3 activity, caused directly by changes in AMY3 protein abundance.

##### Complete Loss of AMY3 Partially Suppresses the ss4 and ss3 ss4 Mutant Phenotypes

We hypothesized that the pleiotropic reduction of AMY3 levels in *ss4* and *ss3 ss4* may result from a regulatory mechanism, whereby a reduced capacity for starch degradation promotes starch synthesis, compensating for the genetic impairment. To further investigate this, we crossed the *amy3* knock-out mutant allele (17, 20, 21) into the *ss4* and *ss3 ss4* mutant backgrounds. Homozygous *amy3 ss4* and *amy3 ss3* double mutants, as well as homozygous *amy3 ss3 ss4* triple mutants, were selected in the F_2_ generation by PCR-based genotyping.

We then examined the growth phenotype of these plants compared with their parents and the wild type. Under our growth conditions, the *ss4* mutant had reduced growth compared with the wild type and had pale leaves (Fig. 2*A*). The *ss3 ss4* mutant was severely stunted in growth and even paler than *ss4*. In contrast, the *amy3* single mutant was indistinguishable from the wild type. These phenotypes were comparable with those observed in previous studies (13, 14, 16, 17). Interestingly, the *amy3 ss4* double mutant did not display the characteristic paleness of the *ss4* parent. It had larger rosettes than *ss4* but was still slightly smaller than the wild type and the *amy3* single mutant. Although the *amy3 ss3 ss4* triple mutant was clearly compromised in growth compared with the wild type, it grew much larger than the *ss3 ss4* double mutant and was less pale. Therefore, the mutation of AMY3 partially suppressed the growth and morphological phenotypes of the *ss4* and *ss3 ss4* mutants.

To assess starch levels in these mutants, rosettes were harvested at the end of the day, and starch within tissues was stained with iodine. There were no obvious differences in staining pattern between the *amy3* single mutant and the wild type (Fig. 2*B*). The *ss4* mutant, as previously documented, stained much darker in older leaves than in younger leaves (15). In contrast, the staining pattern of the *amy3 ss4* mutant was indistinguishable from the wild type. The *amy3 ss3 ss4* mutant also stained positively for starch in all leaves. Although this staining was not strong, it differed from *ss3 ss4*, which failed to stain for starch at all.

We quantified the starch content of these mutants at the end of day and end of night (Table 1). Although the end-of-day starch content in *ss4* was ≈70% of that in the wild type, the mutant retained significantly more starch than the wild type at the end of night. Consistent with the iodine staining, the *amy3 ss4* mutant had significantly higher starch content relative to both *ss4* and the wild type at both time points (*p* < 0.05), accumulating ≈10% more starch than the wild type at the end of the day. Although the *ss3 ss4* mutant was virtually starchless (≈3% of the wild type starch content), the *amy3 ss3 ss4* mutant accumulated measurable levels of starch during the day (≈12% of the wild type starch content), which was degraded completely during the night. The loss of AMY3 can therefore increase starch content in the *ss4* background and restore starch synthesis in *ss3 ss4* background, suggesting that AMY3 is repressing starch accumulation in those mutants. These findings are particularly intriguing because the *amy3* mutation alone has little impact on starch content.

**TABLE 1:** **Starch content of *amy3* multiple mutants** The values for starch content at end of day and end of night time points are the means ± S.E. from *n* = 5–6 individual plants.

Genotype	Starch content	
	End of day	End of night
Col	7.02 ± 0.45	0.27 ± 0.07
*amy3*	6.09 ± 0.61	0.46 ± 0.05*^a^*
*ss4*	4.83 ± 0.25	1.20 ± 0.15
*amy3 ss4*	7.72 ± 0.45*^a^*	2.02 ± 0.19*^a^*
*ss3 ss4*	0.20 ± 0.06	0.18 ± 0.02
*amy3 ss3 ss4*	0.86 ± 0.10*^a^*	0.15 ± 0.03

*^a^* Value is significantly different to that in the corresponding genetic background without the *amy3* mutation (i.e.: *amy3 vs.* Col, *amy3 ss4 vs.* ss4, and *amy3 ss3 ss4 vs. ss3 ss4*) under a two-tailed *t* test at *p* < 0.05.

##### amy3 ss4 Mutants Accumulate Less ADP-glucose

**FIGURE 3.**
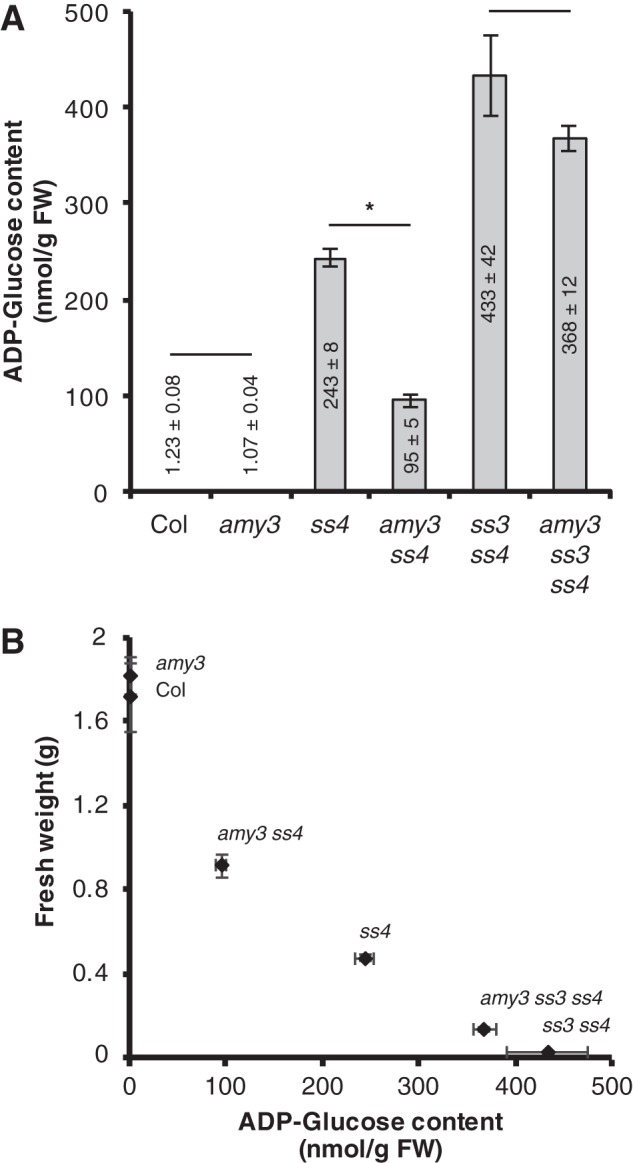
***amy3 ss4* accumulates less ADP-glucose than *ss4*.** *A*, ADP-glucose content was determined using UHPLC-MS/MS. Entire rosettes of 4-week-old plants were harvested at the end of the day. Metabolites were extracted using chloroform/methanol. The values represent means ± S.E. (*n* = 4). An *asterisk* represents significant difference under a two-tailed *t* test at *p* < 0.05. *B*, correlation between ADP-glucose content (as measured in *A*) and plant growth. The fresh weight values are those shown in Fig. 2*A*.

It has been proposed that *ss4* and *ss3 ss4* mutants are pale and growth-compromised because of the accumulation of ADP-glucose, the substrate for starch synthases (16). Given that the *amy3 ss4* mutant was not noticeably pale and given the improved growth of *amy3 ss3 ss4* over *ss3 ss4*, we measured the ADP-glucose content of these mutants. We extracted metabolites from plants harvested at the end of day and assayed ADP-glucose using liquid chromatography coupled with mass spectrometry. The ADP-glucose pool was highest in the *ss3 ss4* mutant, accumulating 433 nmol/g FW, and lowest in the wild type and in *amy3*, where levels were around 1 nmol/g FW (Fig. 3*A*). *ss4* plants accumulated 243 nmol/g FW ADP-glucose. The *amy3 ss4* mutant had significantly less ADP-glucose than *ss4* (a reduction of 60%). Despite the improved growth of *amy3 ss3 ss4* compared with *ss3 ss4*, we did not observe a significant difference in ADP-glucose content between the two mutants. However, there was overall a strong negative correlation between ADP-glucose content and plant growth determined by fresh weight (*r* = −0.97) (Fig. 3*B*). Visibly pale phenotypes were observed in the three mutants with the highest ADP-glucose content (*ss4*, *ss3 ss4*, and *amy3 ss3 ss4*) (Fig. 2). These data suggest that there is a threshold in the amount of adenylates that can be sequestered into ADP-glucose without having a detrimental effect on the chloroplasts. Indeed, it was previously shown that the *aps1 ss3 ss4* mutant accumulates 30–60 nmol/g FW ADP-glucose, only slightly lower than the levels we measured in the *amy3 ss4* mutant (16). The *aps1 ss3 ss4* line does not have a pale phenotype and grows at a similar rate to the *aps1* parent (the *aps1* mutant lacks the small subunit of ADP-glucose pyrophosphorylase).

##### Loss of AMY3 Increases the Number of Starch Granule Initiations in the ss4 Background

The *ss4* mutant has dramatically reduced granule number per chloroplast. Because *amy3 ss4* mutants accumulated more starch than *ss4* and less ADP-glucose, we investigated whether this resulted from the production of larger starch granules or an increased number of granule initiations in the double mutant. To test this, we embedded leaf tissue from young and old leaves of wild-type and mutant plants in resin. Thin sections were cut and stained in toluidine blue to visualize starch granules using light microscopy.

**FIGURE 4.**
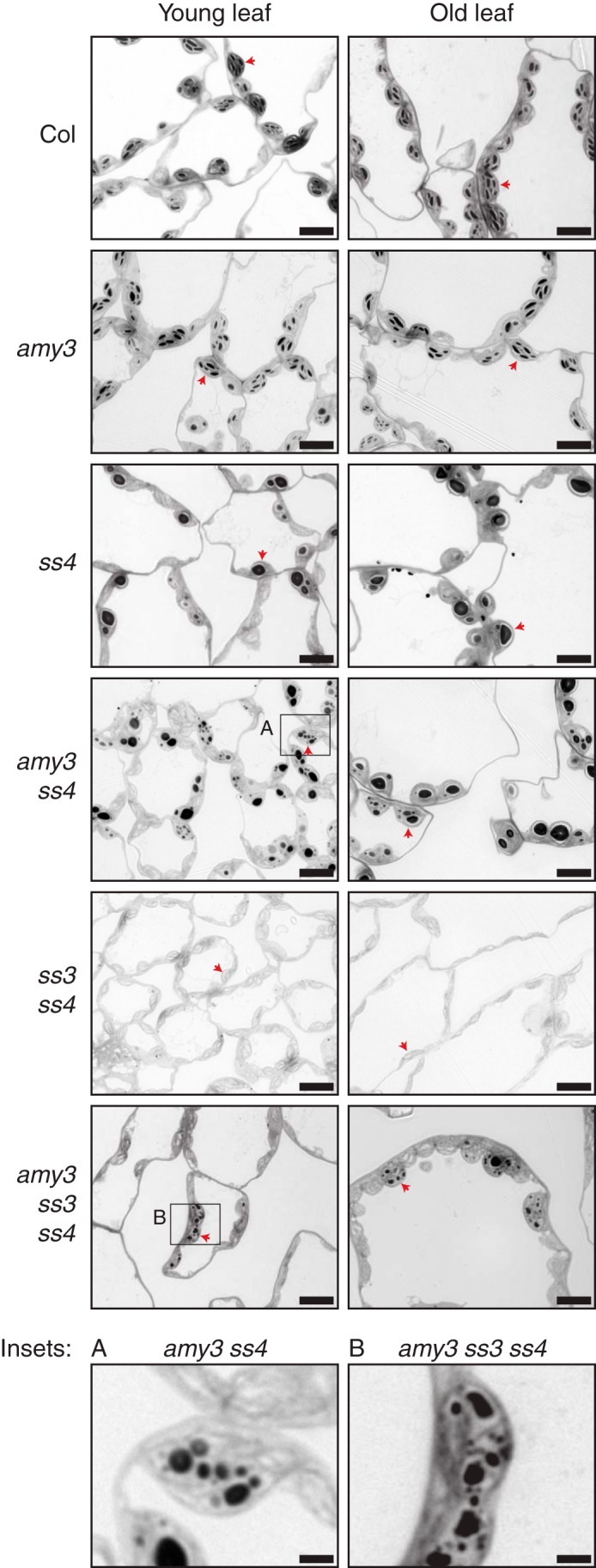
**Loss of *amy3* increases granule number per chloroplast in *ss4* and *ss3 ss4*.** Leaf tissue from young and old leaves was harvested at the end of the day and fixed prior to embedding in resin. Young and old leaves were defined as indicated in Fig. 2*A*. Thin sections were cut, and starch was stained with toluidine blue prior to visualization by light microscopy. *Red arrows* indicate representative chloroplasts. *Bars*, 10 μm for *main panels* and 2 μm for *insets*.

Chloroplasts sections in mesophyll cells of wild-type plants had numerous flattened starch granules (Fig. 4). No obvious differences in granule number were visible between young and old leaves. Chloroplast sections in *ss4* mutants had either zero, one, or rarely two round granules. However, chloroplast sections with no starch granules were observed more frequently in younger leaves than old leaves. In *amy3 ss4* chloroplast sections, many smaller round granules were present, in addition to some large starch granules similar to those observed in *ss4*. Some chloroplast sections in *amy3 ss4* only contained many small granules (Fig. 4, *inset A*), which was never observed in the *ss4* single mutant. Unlike *ss3 ss4* chloroplasts, which contained no starch, some chloroplast sections in the *amy3 ss3 ss4* triple mutant contained numerous, small round starch granules (Fig. 4, *inset B*). The majority of chloroplast sections of this mutant, however, still contained no starch. The loss of AMY3 can therefore increase the frequency of granules in both *ss4* and *ss3 ss4* mutants. No obvious difference was observed between the wild type and *amy3* single mutant.

**FIGURE 5.**
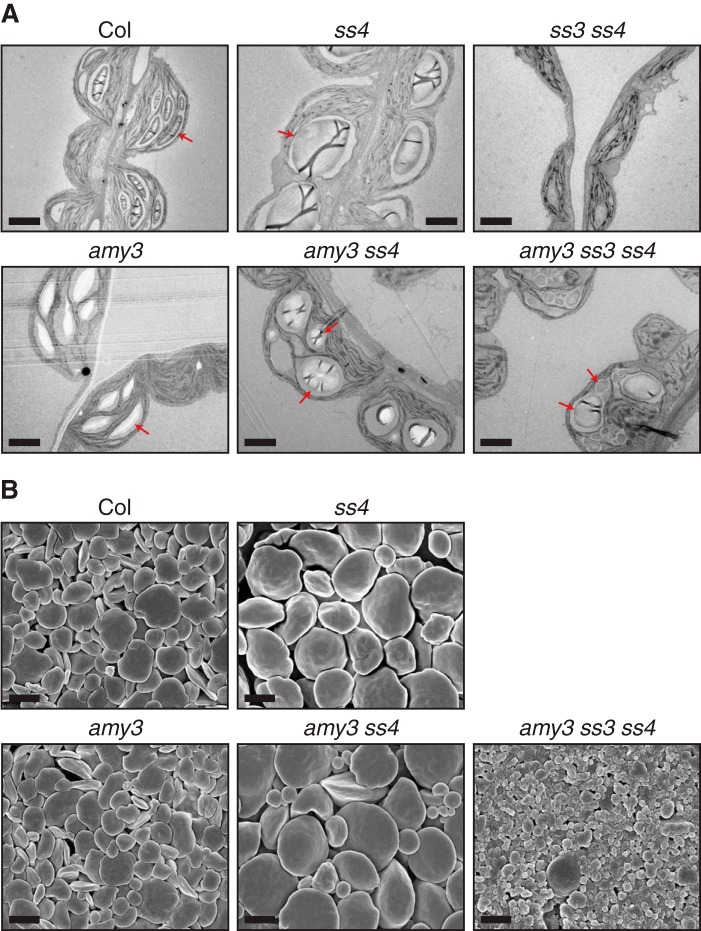
**Alteration of granule morphology in *ss4* is independent from granule number.** *A*, observation of granule morphology using transmission electron microscopy. Leaf sections were prepared from tissue harvested at the end of the day. *Red arrows* indicate representative starch granules. *Bars*, 2 μm. *B*, starch granules purified from *Arabidopsis* rosettes at the end of the day and examined using scanning electron microscopy. *Bars*, 2 μm.

Despite the numerous granules in *amy3 ss4* and *amy3 ss3 ss4* chloroplasts, we noted that the shape of their granules still had the distinct round morphology typical of the *ss4* mutant rather than the flattened discoid shape of wild-type starch. To further investigate these differences in granule morphology, we used electron microscopy. First, we generated transmission electron microscopy images of chloroplasts in these mutants. The *amy3 ss4* mutant contained rounded granules like the *ss4* parent (Fig. 5*A*). Although some granules in *amy3 ss3 ss4* had round morphology similar to *amy3 ss4* granules, there were also some smaller granules with irregular appearance. The flattened discoid granules were only observed in the wild type and *amy3* mutant. We also purified starch from rosettes and viewed it using scanning electron microscopy. Starch granules from *amy3 ss4* and *ss4* had distinct round morphology and were larger than wild-type granules (Fig. 5*B*). However, consistent with the light microscopy images, there was a distinct subpopulation of smaller granules in *amy3 ss4*. Starch from *amy3 ss3 ss4* was mainly composed of very small granules, although some larger ones could be observed. The loss of SS4 therefore causes a distinct change in granule morphology that is independent from the number of granules initiated per chloroplast.

**FIGURE 6.**
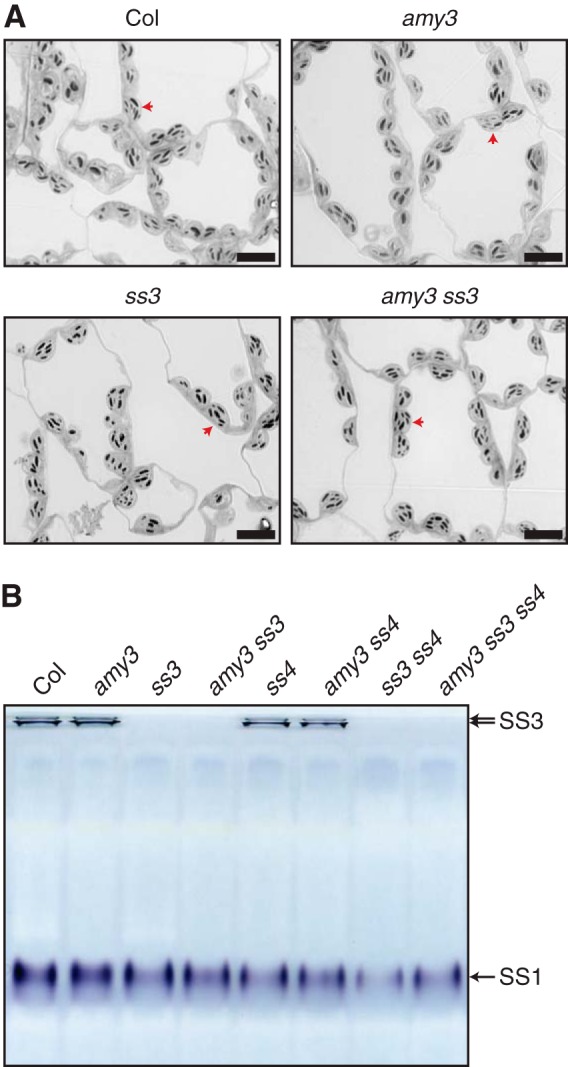
**SS3 is not required for granule initiation in the presence of SS4.** *A*, light micrographs of starch granules in *ss3* and *amy3 ss3* mutant chloroplasts. Leaf sections were prepared from young leaves harvested at the end of the day. *Red arrows* indicate representative chloroplasts. *Bars*, 10 μm. *B*, native PAGE analysis of starch synthase activity. Soluble proteins were extracted from leaves harvested 4 h into the day and were loaded onto gels containing 0.15% amylopectin on an equal fresh weight (2.7 mg) basis. Starch synthase activity bands were visualized by incubating the gel in the presence of ADP-glucose, followed by staining with iodine.

Because the *amy3 ss3 ss4* mutant had a stronger phenotype than *amy3 ss4*, we further investigated the influence of SS3 on granule initiation and morphology. We observed chloroplast sections of *ss3* and *amy3 ss3* mutants by light microscopy, but the number and shape of the granules in these mutants were indistinguishable from the wild type (Fig. 6*A*). This suggests that in the presence of SS4, SS3 is dispensable in granule initiation. We also assessed whether there were pleiotropic changes in SS3 levels in *amy3 ss4* mutants using native PAGE gels stained for starch synthase activity. We could detect both SS1 and SS3 activities, but both were similar between *amy3 ss4* and the wild type (Fig. 6*B*). It is therefore unlikely that the *amy3 ss4* mutant accumulates more starch than *ss4* because of pleiotropic changes in the activity of other starch synthases.

## Discussion

### 

#### 

##### AMY3 Interferes with the Granule Initiation Process

It is clear from genetic evidence that SS4 plays a key role in granule initiation in *Arabidopsis* (13). The *ss4* mutant has a strong reduction in starch content and granule number per chloroplast. Furthermore, the *ss3 ss4* double mutant is essentially starchless (14). The presence of SS1 and SS2 alone was therefore thought to be insufficient for generating any crystalline starch (14). Our work demonstrates that the phenotypes of *ss4* and *ss3 ss4* mutants arise not only from defects in granule synthesis but also from glucan degradation. In particular, we show that the α-amylase, AMY3, strongly influences granule formation and granule number per chloroplast in these mutants. Abolishing AMY3 activity in the *ss4* background increased total starch content, as well as the number of granules per chloroplast. Abolishing AMY3 activity in the *ss3 ss4* background partially restored starch synthesis (Figs. 4 and 5). Granule initiations in both *ss4* and *ss3 ss4* mutants are therefore limited by the presence of AMY3.

We suggest that SS4 plays a critical role by allowing granule initiation to proceed in a way that prevents premature hydrolysis. In the absence of SS4, AMY3 may interfere with the granule initiation process by degrading soluble glucan primers that precede crystalline starch. This interference appears to have a negligible influence in wild-type plants, because the *amy3* single mutant has a normal granule number per chloroplast (Fig. 4). Hence, the presence of SS4 in the wild type may prevent AMY3 interference, perhaps by elaborating soluble pre-starch primer glucans into crystalline structures that evade premature degradation. We expect that SS4 would fulfill this role in conjunction with branching and debranching enzymes. Once crystallinity is “seeded” by these enzymes, the other SSs can proceed to synthesize amylopectin structure and produce the body of the starch granule. Interestingly, AMY3 activity *in vitro* is much higher against soluble substrates than against crystalline starch (20), suggesting that it is more likely to interfere with the granule initiation process before crystallinity develops. This specific degradation of glucan primers before the formation of crystalline starch would limit the primers available for the other SSs, resulting in the observed accumulation of ADP-glucose (Fig. 3). Additionally, no accumulation of glucan degradation products (*e.g.* maltose and other maltooligosaccharides) was reported in *ss4* (13), an observation that we confirmed (data not shown). Mutants that simultaneously degrade large amounts of starch as it is being made during the day contain higher levels of maltose (10, 22). In *ss4*, the presence of large amounts of ADP-glucose and the absence of large amounts of maltooligosaccharides support the idea that AMY3 inhibits the synthesis of starch granules by degrading glucan primers. Because we would expect a very low abundance of these primers, their degradation would not lead to a measurable accumulation of maltooligosaccharides.

A notable difference between our *amy3 ss4* mutant and the *gwd ss4* (also called *ss4 sex1*) double mutant (reported in Ref. 15) further supports the idea that AMY3 interferes with granule initiation before crystallinity develops. GWD is a glucan, water dikinase that is essential for proper degradation of starch granules. By phosphorylating glucan chains at the crystalline granule surface, GWD is thought to disrupt glucan packing and increase their accessibility to β-amylases (2, 23). Phosphorylation by GWD is thought only to be required for the degradation of crystalline granules (23), and the recombinant protein preferentially phosphorylates crystalline substrates *in vitro* (24). Although older leaves of *gwd ss4* contained high levels of starch, younger leaves contained very little starch, resembling the *ss4* parent. This suggests that blocking starch phosphorylation has no effect on starch granule initiation in younger leaves of *ss4*. By contrast, the *amy3 ss4* mutant was able to synthesize starch in the entire rosette, including the younger leaves (Fig. 2). Together, these findings suggest that in the younger leaves of *ss4*, AMY3 degrades glucan primers and consequently limits granule initiation. GWD does not contribute to this degradation because at this stage, nothing crystalline has been formed.

AMY3 interferes not only with starch granule initiation, but also with other aspects starch granule synthesis if the formation of crystalline glucan is inefficient. A related case is that of the *Arabidopsis* quadruple mutant lacking all debranching enzymes (*isa1 isa2 isa3 lda*), which produces a soluble, glycogen-like polymer in place of starch granules. Introducing the *amy3* mutation in this background (*i.e.* generating a quintuple mutant) restored insoluble granule formation (10). In addition, the quintuple mutant accumulated more soluble glucans than the quadruple mutant. This was interpreted to mean that, in the quadruple mutant, AMY3 actively degrades soluble glucans, hindering the development of crystallinity (10).

Overall, our work re-emphasizes that starch degradation enzymes are active in starch synthesis mutants and the interpretation of phenotypes must take this into account. We speculate that mutating other glucan-hydrolyzing enzymes in *ss4* or *ss3 ss4* may also increase starch content, particularly if those enzymes can also degrade the soluble precursors of granules. The ISA1-ISA2 isoamylase involved in partially debranching amylopectin to promote its crystallization has been shown to degrade some branched substrates, such as glycogen or aberrant amylopectin, suppressing production rather than promoting crystallization (25, 26). Furthermore, increased numbers of granules have been noted in some *isa* mutants alongside the accumulation of soluble phytoglycogen (27–29). Other enzymes may include the β-amylase-3 (BAM3) and isoamylase-3 (ISA3), both of which are important for night-time starch degradation. The loss of function in these enzymes lead to starch excess phenotypes (30, 31). However, our results with AMY3 are particularly interesting because it is not required for night-time starch degradation under normal growth conditions, and *amy3* single mutants turn over starch similarly to the wild type (17) (Fig. 2 and Table 1).

##### An Updated Model of Granule Initiation in Chloroplasts

It was previously speculated that SS4 may “control” granule numbers in the chloroplast. This was inferred from the drastic reduction in granule number in *ss4* chloroplasts. Because this phenotype is highly dependent on AMY3 activity, we suggest that it is unlikely SS4 alone exerts strict control over granule number.

Nevertheless, SS4 does play a unique role, because it appears to act on primer glucans in a way that prevents premature hydrolysis by AMY3. SS3 appears to have some activity on glucan primers, because it is able to initiate some granules in the *ss4* mutant, but it is not efficient because it cannot overcome the glucan degradation by AMY3. It is only in the absence of AMY3 that SS3 can initiate granules frequently, indicated by the fact that the *amy3 ss4* mutant accumulated much more starch than *amy3 ss3 ss4*. However, in the presence of SS4, SS3 is dispensable for initiating granules (Fig. 6).

It is unclear which properties of SS4 allow it to act on primers efficiently. It may be related to substrate specificity and kinetics. It could also relate to specific features of the protein, such as the large N-terminal extension that contains coiled coils (5, 32), which are typically implicated in protein-protein interaction (33). The N-terminal has been recently shown to play a role in the localizing the protein to thylakoid membranes within the chloroplast, through interaction with plastidial fibrillins (34). However, SS4 may also bind to partner enzymes that modify or interact with the substrate in a way that promotes crystallinity (*e.g.* branching/debranching enzymes). SS4 can form dimers, which may increase the efficiency of crystalline glucan formation (34). It could also interact with other, as-yet unidentified protein factors that control granule number within each chloroplast.

We suspect that the primer glucans are generated from two distinct sources: first from the dynamic elongation of malto-oligosaccharides in the chloroplast stroma and second from starch degradation. The stochastic generation of malto-oligosaccharides may prime the synthesis of the first starch granules within each chloroplast. All starch synthase isoforms can elongate malto-oligosaccharides into longer glucan primers that have a higher propensity to develop crystallinity (14, 35). The α-glucan phosphorylase can also synthesize malto-oligosaccharides *in vitro* (36). The presence of numerous enzymes capable of generating malto-oligosaccharides in the stroma may explain why individual mutations in the other starch synthases or in the plastidial isoform of phosphorylase do not appear to affect granule number (14, 37). Once a granule is initiated within the chloroplast, the degradation of this starch could provide another potential source of primers. This may explain the very uneven distribution of granules among chloroplasts in the *amy3 ss3 ss4* mutant; although the majority of chloroplasts have no starch, those that do have starch contain many granules. In the *ss4* mutant, the majority of glucan primers produced initially through malto-oligosaccharide elongation may be degraded by AMY3, except for a minority that is elongated by SS3. The degradation of starch in this mutant may also generate potential primers, but the degradation of these by AMY3 also suppresses the formation of more granules.

A recent study suggested that the presence of SS3 and SS4 may be less critical for granule initiation in cereal endosperm than in leaves. A 43% reduction in grain starch content relative to the wild type was observed in a rice mutant lacking major isoforms of SS3 and SS4, showing that the majority of granules were initiated in the absence of either isoform (38). It should be noted that other isoforms of SS3 and SS4 are present in this mutant that could contribute to granule initiation, although their expression may be relatively low in the endosperm (39). However, our study raises a further possibility that the mutant can accumulate starch in the endosperm because of differences in how starch-degrading enzymes are regulated in that tissue. Indeed in developing wheat grains, AMY3 protein is present in pericarp and aleurone layers but is not detectable in endosperm tissue (40). Further information will be necessary to determine whether SS3 and SS4 play similar roles in granule initiation in both leaf and endosperm.

##### SS4 Plays a Dual Role in Initiating Starch Granules and Influencing Granule Shape

In addition to the reduction in granule number, *ss4* mutants have starch granules with a round appearance, distinct from the discoid appearance of wild-type granules (13–15). It was previously unclear whether the alteration in granule shape is due to having fewer granule initiations, whereby a single initiation grows spherically until a round granule is formed, or whether SS4 directly influences granule shape. Interestingly, the overexpression of glycogen synthase from *Agrobacterium tumefaciens*, which can initiate glucan synthesis by self-glucosylatation (41), was able to restore starch biosynthesis in *ss3 ss4* mutants (15). Although this increased granule number per chloroplast, all granules had a round morphology similar to that observed in *ss4*. This shows that round granules can form regardless of the number of granules per chloroplast, implying a direct role for SS4 in controlling granule morphology. Our study supports this idea. Despite the increase in granule number relative to *ss4*, the *amy3 ss4* mutant clearly has round granule morphology that closely resembles its *ss4* parent (Fig. 5). This again suggests that the altered shape of granules in *ss4* is independent from granule number per chloroplast. Further work is required to determine which features of SS4 allow it to influence granule morphology. Interestingly, *ss4* mutants have only slightly altered amylopectin chain length distribution (14), suggesting that the control of granule morphology may be only indirectly related to factors that determine amylopectin structure. It is also unclear how the dual role of SS4 in granule initiation and morphology may be regulated. In *Arabidopsis*, SS4 has been reported to localize to the periphery of starch granules, as well as bound to the thylakoid membrane (14, 42). It is possible that these different localizations may be required for SS4 to play its multiple roles.

Interestingly, SS3 and SS4 also influence granule morphology in the endosperm of rice, where multiple initiations result in compound granules, each composed of several individual polygonal granules. The individual granules from *ss3a* single mutants are more rounded than the polygonal ones of the wild type, and this rounded-granule phenotype is even more accentuated in *ss3a ss4b* double mutants (38, 43). However, it is difficult to assess whether SS3 or SS4 plays the more important role there, because other isoforms (SS3b and SS4a) are still present in these mutants.

##### Regulation of AMY3 Abundance and Activity

We observed a pleiotropic reduction in AMY3 abundance in the *ss4* and *ss3 ss4* mutants. Such a decrease was not observed for the other starch-degrading enzymes assessed on our native PAGE gels. This hints at a regulatory mechanism in plants for specifically reducing AMY3 activity when its presence is detrimental for plant growth. However, this regulation is clearly not sufficient to maintain proper granule initiation in *ss4* and *ss3 ss4*. Residual AMY3 protein is still detectable in these mutants by immunoblotting, and AMY3 transcript levels in these mutants are not lower than in wild type (Fig. 1). This suggests that AMY3 is continuously expressed but does not accumulate in the mutants. The residual AMY3 protein and activity is evidently important, because the total abolition of AMY3 greatly alleviates the phenotypes of the mutants. It is tempting to speculate that the *ss4* and *ss3 ss4* phenotypes would be much more severe if AMY3 protein levels were not down-regulated via this as-yet unexplored post-transcriptional mechanism.

A similar pleiotropic reduction in AMY3 abundance has been observed in the *sex4* mutant (17, 44). The *SEX4* locus encodes for a glucan phosphatase essential for proper starch degradation. Like in the *ss4* and *ss3 ss4* mutants, AMY3 is still active in the *sex4* mutant despite reduced abundance; the mutant accumulates phosphorylated soluble oligosaccharides that are released by the AMY3 (21). Abolition of AMY3 in the *sex4* background greatly reduced the accumulation of these phosphorylated oligosaccharides. The stimulus and mechanism by which AMY3 abundance is controlled are yet unknown. However, we note that *sex4* mutants also produce large round granules, conspicuously similar in appearance to those of *ss4* (45); an unexplained aspect of the *sex4* mutant phenotype. It would be interesting to investigate whether SS4 activity is also pleiotropically altered in *sex4* mutants.

## Experimental Procedures

### 

#### 

##### Plant Materials and Growth Conditions

*Arabidopsis thaliana* plants were grown in soil in a controlled environment chamber (Percival AR-95L, CLF Plant Climatics; or Kälte 3000) providing a 12-h light/12-h dark cycle, with a light intensity of 150 μmol photons m^−2^ s^−1^, a constant temperature of 20 °C, and a relative humidity of 65%. The following T-DNA insertion mutants were used in this study: *ss4* (*ss4-1* as described in Refs. 13 and 14; GABI_290D11), *ss3 ss4* (as described in Ref. 14; SALK_065732 × GABI_290D11), and *amy3* (*amy3-2* as described in Ref. 17; SAIL_613D12). All lines are in the Columbia accession. For generating *amy3* multiple mutants, *amy3* plants were crossed with the *ss3 ss4* mutant. Homozygous *amy3 ss3*, *amy3 ss4*, and *amy3 ss3 ss4* plants were isolated in the F_2_ generation using PCR-based genotyping, carried out as previously described (14, 21).

##### Native PAGE and Immunoblotting

Activities of AMY3 or SS isoforms in protein extracts from leaves were visualized by native PAGE as previously described (5, 20). The gels were loaded according to an equal fresh weight of plant material per lane. For immunoblot detection of AMY3, proteins were extracted from *Arabidopsis* leaves by homogenization in protein extraction medium (40 mm Tris-HCl, pH 6.8, 5 mm MgCl_2_, and Complete Protease inhibitor mixture) at a ratio of 1 ml of medium/100 mg of tissue. Insoluble material was removed by centrifugation. Proteins in the supernatant were separated by SDS-PAGE (loaded on equal fresh weight basis) and subsequently transferred onto PVDF membranes using standard procedures. AMY3 antiserum, generated previously by immunizing rabbits with recombinant AMY3 protein (17), was used in conjunction with secondary antibodies conjugated to the 800CW infrared fluorescent dye (excited at 800 nm). Actin was detected concurrently in the same blots as a loading control using a monoclonal antibody raised in mouse (Sigma A0480) together with secondary antibodies conjugated to 680RD dye (excited at 700 nm). Proteins were detected via infrared fluorescence using the Odyssey CLx system (Li-Cor).

##### Quantification of AMY3 Transcript by Quantitative RT-PCR

Total RNA was extracted from entire rosettes harvested at the indicated time points using the RNeasy plant RNA purification kit with DNase treatment (Qiagen). Reverse transcription was carried out on 2 μg of total RNA using the RevertAid reverse transcriptase kit (Thermo-Fisher Scientific). We then conducted quantitative RT-PCR to quantify AMY3 transcript levels using the Fast SYBR Green master mix together with a 7500 Fast real time PCR system (Applied Biosystems), with the following primers: forward, 5′-TGCTTACATCCTAACTCATCC-3′; and reverse, 5′-CTCTTGTCTATATTCACCTCACTC-3′. Transcript levels were calculated relative to the *YLS8* reference gene, which was amplified with the following primers: forward, 5′-TTACTGTTTCGGTTGTTCTCCATTT-3′; and reverse, 5′-CACTGAATCATGTTCGAAGCAAGT-3′.

##### Starch Staining and Quantification

For staining with iodine, rosettes harvested at the end of the day were clarified in 80% (v/v) ethanol before staining in Lugol's iodine solution (Sigma), as described previously (46). Excess iodine was removed by washing in water prior to imaging. Starch was quantified as previously described (46). Briefly, entire rosettes were homogenized in perchloric acid. Insoluble material was collected by centrifugation, and starch therein was digested to glucose using α-amylase and amyloglucosidase. Glucose was quantified using the hexokinase/glucose-6-phosphate dehydrogenase method.

##### ADP-Glucose Quantification

Plant material was harvested in the light at the end of the day and immediately frozen in liquid N_2_. Care was taken to ensure that the leaves were not shaded prior to rapid freezing. The tissue was ground and extracted using chloroform-methanol as previously described (47). Extracted metabolites were dried in a vacuum centrifuge, dissolved in water and filtered through a 0.2-μm cellulose membrane. The samples were analyzed using a UHPLC-MS/MS consisting of a 1290 Infinity UHPLC (Agilent) with an Acquity T3 end-capped reverse phase column (Waters), coupled to a QTRAP 5500 triple quadrupole MS (AB Sciex). ADP-glucose was quantified against a standard curve prepared using commercial ADP-glucose (Sigma-Aldrich A0627)

##### Examination of Starch Granule/Chloroplast Morphology

Light microscopy and transmission electron microscopy analyses were carried out as previously described (5). For scanning electron microscopy, starch was extracted as described in (5) and was visualized using a Leo 1530 scanning electron microscope (Zeiss).

## Author Contributions

D. S., S. S., and S. C. Z. conceived and designed the experiments. D. S., K.-J. L., and M. S. performed the experiments. D. S. and S. C. Z. wrote the paper. All authors analyzed the results and approved the final version of the manuscript.

## Footnotes

2 The abbreviation used is:

SS: starch synthase.
